# Implementation of a Framework for Healthy and Diabetic Retinopathy Retinal Image Recognition

**DOI:** 10.1155/2020/4972527

**Published:** 2020-05-13

**Authors:** Oluwatobi Noah Akande, Oluwakemi Christiana Abikoye, Aderonke Anthonia Kayode, Yema Lamari

**Affiliations:** ^1^Computer Science Department, Landmark University, Omu-Aran, Kwara, Nigeria; ^2^Computer Science Department, University of Ilorin, Ilorin, Kwara, Nigeria; ^3^Computer Science Department, University of Carthage, Tunis, Tunisia

## Abstract

The feature extraction stage remains a major component of every biometric recognition system. In most instances, the eventual accuracy of a recognition system is dependent on the features extracted from the biometric trait and the feature extraction technique adopted. The widely adopted technique employs features extracted from healthy retinal images in training retina recognition system. However, literature has shown that certain eye diseases such as diabetic retinopathy (DR), hypertensive retinopathy, glaucoma, and cataract could alter the recognition accuracy of the retina recognition system. This connotes that a robust retina recognition system should be designed to accommodate healthy and diseased retinal images. A framework with two different approaches for retina image recognition is presented in this study. The first approach employed structural features for healthy retinal image recognition while the second employed vascular and lesion-based features for DR retinal image recognition. Any input retinal image was first examined for the presence of DR symptoms before the appropriate feature extraction technique was adopted. Recognition rates of 100% and 97.23% were achieved for the healthy and DR retinal images, respectively, and a false acceptance rate of 0.0444 and a false rejection rate of 0.0133 were also achieved.

## 1. Introduction

In this era of cutting-edge technology, the demand for a reliable security system is increasing like that of biometric security systems, which employ unique human physical, chemical, and behavioral traits in identifying and authenticating the user of a biometric system. Among these traits, human retina is the most stable, reliable, and secured biometric trait for human authentication and verification [1–3]. Its nonexposure to the environment and swiftness of decay in dead people make it difficult to forge. A retinal biometric system takes into account these properties as well as the invariant structure of patterns present on retinal blood vessels to establish an individual's identity [4]. However, human retina is not resistive to certain diseases including diabetic retinopathy (DR), hypertensive retinopathy, glaucoma, high blood pressure, autoimmune deficiency syndrome, arteriosclerosis, and cardiovascular disease [2]. DR being prevalent among these diseases has been shown to cause measurable changes in the retinal blood vessels' diameter [5], branching angles, width, tortuosity, and length [6]. This has been revealed to have an adverse effect on the identification process and the eventual accuracy of the retinal biometric system [7–9].

Diabetic retinopathy is a common microvascular complication of diabetes [10], which is capable of damaging retinal blood vessels and could further lead to total blindness of the eye. Complications of DR are seen in the disorder of the retinal vasculature that causes a progressive damage to retinal blood vessels which could eventually lead to a partial or total loss of vision and blindness [11, 12]. The presence of DR could also lead to changes in blood vessel structure and vessel distribution which in turn could result in new vessel growth [13]. Also, changes in the blood vessel diameter are direct indicators of retinal vasculature abnormality traceable to DR [5]. Furthermore, the retina infected by DR shows signs of lesions such as microaneurysms, cotton wool spots, exudates, macular edema, and hemorrhages [14]. All these symptoms of DR as seen in retinal images could have adverse effects on the recognition accuracy of retina recognition systems [6, 15, 16]. Therefore, researchers have identified the feature extraction stage as an important stage that could improve the recognition accuracy of biometric recognition systems [1, 3, 17, 18]. Though existing works have proposed several novel feature extraction techniques for healthy or unhealthy retinal images, this study presents a framework that could accommodate both healthy and DR retinal images. Every input retinal image was examined for the symptoms of DR which are majorly exudates. Lesion-based features were then extracted from retinal images with exudates. In addition, blood vessels were also segmented from DR retinal images.

## 2. Related Works

Several literature studies have performed the use of retina images for recognition using different techniques. Some have employed healthy retinal images to validate their techniques while some have used unhealthy retinal images. Prior to the recognition task, different components of the retina such as blood vessels or the optic disk could be segmented while a number of features could also be extracted. Bifurcation points of retinal blood vessels were employed for human identification in [19]. Skeletonization process was used to measure the degree of connectivity of the candidate pixels in the extracted blood vessels. High recognition and low error rate were achieved. Similarly, retina crossover points and vascular branches were also employed for human recognition in [20]. Geometric hashing was used to compute invariant features from the detected crossover points and vascular branch. A 100% detection accuracy was achieved when 165 retinal images were used to validate the proposed technique. In the same way, the authors of [21] employ Fractal Dimension (FDM), Morphological Segmentation and Branching Points (MSBP), and Watershed and SVM (WSM) methods for human identification purpose. FDM computes the fractal dimension of the retina blood vessel image using box-counting algorithm while MSBP employs the bifurcation, branch, and crossover points. WSM uses the wavelet features computed from the segmented retinal blood vessels for identification. The identification accuracy of 96%, 94%, and 92% was recorded with the WSM, MSBP, and FDM techniques, respectively. A five-layer Adaptive Neuro-Fuzzy Inference System (ANFIS) was employed for the retinal recognition system in [22]. Principal Component Analysis (PCA) was used to extract the retina features. With a feature length of 101, an average recognition accuracy of 96% was achieved.

Also, Point Set Matching was employed for retinal recognition in [23]. The optic disc (OD) was localized and extracted using active contour technique; afterward, edge pixels of the OD blood vessel map were employed for human retina recognition. However, a recognition accuracy of 90.21% was achieved. A retinal recognition system that uses retinal blood vessel topology for human recognition was proposed in [24]. A characteristic feature matrix computed from the topological features was used for the recognition task. A recognition accuracy of 95% was achieved when the technique was evaluated. Furthermore, the authors of [14] employed vessel bifurcations and crossover points computed from retina blood vessels for human recognition. Afterward, Scale Invariant Feature Transform (SIFT) descriptors were computed from the features extracted. The principal component analysis was used to reduce the large dimension of the descriptors before a sparse classifier was used for recognition purposes. A classification accuracy of 94.64% was achieved. A technique that does not depend on the much employed preprocessing, segmentation, and feature extraction stages of recognition systems was proposed in [25]. Computed Structural Similarity Index (SSI) of retinal images was used to uniquely differentiate them from other retinal images. The SSI computes the structure, contrast, and luminance of each retinal image as these values are unique for different retinal images. The proposed technique achieved a recognition rate of 99.97% and a faster execution time. On the contrary, the authors of [18] proposed two techniques for retina image recognition. The first technique extracts vascular and nonvascular features from segmented retinal blood vessels while the second technique extracts structural features from retinal blood vessels. The first technique recorded a recognition rate of 100% and execution time of 127.8915 s while the second technique recorded a recognition rate of 92.5% and execution time of 70.9537 s. From the literature reviewed so far, it was observed that retina blood vessel is the principal component from which features are being extracted for recognition purposes. Also, literature has revealed that certain eye diseases such as glaucoma, hypertensive retinopathy, diabetes retinopathy, and cataract could alter or eventually damage the patterns of retina blood vessels. Therefore, this study proposed a technique to extract features from both healthy and diabetic retinopathy retinal images for the purpose of human recognition.

## 3. Materials and Methods

In this section, the methodology behind the proposed technique is explained in detail.

### 3.1. Data Acquisition

Two categories of retinal images were used in training and testing the proposed retinal recognition system. The first category is the retinal images of healthy individuals; these were acquired from public retina databases such as Digital Retinal Image for Vessel Extraction (DRIVE) and High-Resolution Fundus (HRF) Image Database. The second category is the retinal images of DR individuals; these were acquired from public retina databases such as Diabetic Retinopathy Database (DIARETDB1) and Indian Diabetic Retinopathy Image Dataset (IDRiD). DRIVE database contains 40 retinal images obtained at 45° Field of View (FOV) using a Canon CR5 nonmydriatic 3-CCD camera. All the images have a resolution of 768 × 584 pixels with 8 bits per color plane. Thirty healthy retinal images were acquired from DRIVE databases. Similarly, High-Resolution Fundus (HRF) Image Database contains 15 retinal images each of healthy, DR, and glaucomatous patients. These images were captured with a Canon CR-1 fundus camera with a FOV of 45°. 15 healthy retinal images were also acquired from this database. IDRiD contains 516 retinal images captured with Kowa VX-10a retinal fundus camera. The images were captured at 50° FOV and with 4288 × 2848-pixel resolution; 45 DR retinal images were acquired from this database. Furthermore, retinal images from DIARETDB1 were used. The database contains 89 retinal images with pathological signs which are majorly exudates. The images were acquired at 50° FOV with 1500 × 1152-pixel resolution; 30 DR retinal images were acquired from DIARETDB1. Therefore, a total of 45 healthy and 90 DR retinal images were used to evaluate the proposed technique. A summary of the 4 retina datasets used is provided in Table 1.

### 3.2. Image Preprocessing

Most raw retinal images are of low quality. This is a result of the intrusive nature of the retina capturing process. Unlike other biometric traits, the retina is located at the posterior region of the eye which makes its capturing process a difficult one. Much cooperation is needed by users who are expected to position their eyes very close to the lens of the scanning device and remain stationary focusing on a revolving light being emitted by the scanning device. Any movement by the user at this point can interfere with the capturing process and cause the process to be terminated and restarted [18]. Therefore, in an attempt to make the retinal image suitable for analysis, the preprocessing task was carried out to suppress unwanted information while enhancing the needed information. The retinal images acquired were colored images that are made up of red (R), green (G), and blue (B) components; however, red and blue components are very noisy and have low vessel-background contrast [26, 27]. Therefore, the red and green components of the input retinal images were removed while the green component with the best contrast and less noise was extracted and retained. Furthermore, to enhance the intensity of the extracted green component, Mahalanobis distance and CLAHE as proposed in [28] were employed to identify and eliminate the background pixel while enhancing the foreground pixels only. In order to achieve this, the mean *μ*_*N*_ and standard deviation *σ*_*N*_ of the statistical distribution of the intensities in input retinal image N were calculated. Thereafter, a sample mean ${\hat{\mu }}_{N}$ and a sample standard deviation ${\hat{\sigma }}_{N}$ were chosen as the estimators for *μ*_*N*_ and *σ*_*N*_, respectively. The intensities of the image pixel tagged *I* (*x*, *y*) were then compared with the mean intensity using equation (1). If *I* (*x*, *y*) is close to *μ*_*N*_ or if *d*_*M*_ is lower than a specified threshold *t*, then, the pixel belongs to the background image *β*; else, it belongs to the foreground image:

(1) $$\begin{array}{c} F_{\text{enc}}=\left|\frac{I\left(x,y\right)-{\hat{\mu }}_{N}}{{\hat{\sigma }}_{N}}\right|<t, \end{array}$$

where *F*_enc_ is the enhanced foreground image. The preprocessing steps are summarized with the following algorithm:

  Start
  Step 1: input retinal image to preprocess
  Step 2: extract the green component *R*_G_
  Step 3: convert *R*_G_ to grayscale denoted by I
  Step 4: employ Mahalanobis Distance (MD) to separate the foreground image (*F*) from the background image *β*
  Step 5: enhance *F* using CLAHE to give *F*_enc_
  Stop.

### 3.3. Segmentation

A retinal image is composed of several components such as optic disc, blood vessels, macula, and fovea. The process of separating and analyzing these components for effective feature extraction is called segmentation. The component to be separated from others depends on the task at hand. However, the segmentation task becomes more complicated when the retina is infected with diseases and begins to show pathological signs [29]. In retinal images, the major component through which the symptoms of DR can be diagnosed is the blood vessels; therefore, blood vessels will be detected and segmented from the retinal images. In addition, the presence of small yellowish circular patterns (exudates) on the surface of the retina signifies the presence of DR. Hence, for the training purpose, the presence of exudates will be determined from each retinal image and segmented if present. Therefore, the segmentation task in this study will be in two categories: blood vessel segmentation and exudate segmentation.

#### 3.3.1. Blood Vessel Detection and Segmentation

Segmenting blood vessels entails detecting them prior to the identification of their paths. To achieve this, preliminary vessel edge information and vessel map for the input retinal image was obtained using Dempster–Shafer (D-S) edge-based detector proposed in [30]. D-S uses probability-based fusion to merge the outputs of Laplacian of Gaussian (LoG) and canny edge detection filters in determining the continuous paths of a vessel after the starting point has been determined. LoG filter was used to determine which pixel of the input retinal image is an edge pixel using

(2) $$\begin{array}{c} h\left(x,y\right)=\exp\left(-\;\frac{x^{2}+y^{2}}{2\sigma_{N}^{2}}\right),\\ \nabla^{2}h\left(x,y\right)=\left(\frac{x^{2}+y^{2}-\sigma^{2}}{\sigma_{N}^{4}}\right)\exp\left(-\frac{\;x^{2}+y^{2}}{2\sigma_{N}^{2}}\right),\\ g\left(x,y\right)=\nabla^{2}h\left(x,y\right)*F_{\text{enc}}, \end{array}$$

where *F*_enc_ remains the enhanced foreground image, *g* (*x*, *y*) is the output image, *σ*_*N*_ remains the standard deviation, *h* (*x*, *y*) is the 2D Gaussian function, and ∇^2*h* (*x*, *y*) is the LoG filter.

Furthermore, in the edge detection task, after determining the edge pixels in the input retinal image, a canny filter was used to determine the horizontal, vertical, and diagonal edges. The horizontal direction *G*_*y*_ and the vertical direction *G*_*x*_ were computed using equations (3) and (4) respectively:

(3) $$\begin{array}{c} G_{x}=\partial_{x}F_{\text{enc}}\left(x,y\right) \end{array}$$

(4) $$\begin{array}{c} G_{y}=\partial_{y}F_{\text{enc}}\left(x,y\right). \end{array}$$

The resulting edge gradient and direction were determined using

(5) $$\begin{array}{c} G=\sqrt{{\left(\partial_{x}I\left(x.y\right)\right)}^{2}+{\left(\partial_{y}I\left(x.y\right)\right)}^{2}\;}. \end{array}$$

To achieve a more accurate and stable vessel edge detection, a D-S-based edge detector fuses the outputs *g* (*x*, *y*) of the LoG filter and the output *G* of the canny edge. This is referred to as a joint *m*_1_⊕*m*_2_, where *m*_1_ and *m*_2_ are the outputs of LoG and canny edge filter, respectively. The joint *m*_1_⊕_2_ was obtained using equation (6) while the conflicting events caused by LoG and canny filter were removed using equation (7). The basic probability mass “K” associated with the conflicts was calculated using equation (8):

(6) $$\begin{array}{c} m_{1}⊕m_{2}\left(A\right)=\frac{\sum_{B\cap C=A}m_{1}\left(B\right)m_{2}\left(C\right)}{1-K}, \end{array}$$

(7) $$\begin{array}{c} {\left(m_{1}⊕m_{2}\right)}_{\left(\ell \right)}=0, \end{array}$$

(8) $$\begin{array}{c} K=\underset{B\cap C=\ell }{\sum }m_{1}\left(B\right)m_{2}\left(C\right). \end{array}$$

*A*, *B,* and *C* are the event set produced by the D-S fusion, LoG filter, and canny edge filter, respectively. Any edge detection algorithm could detect an edge or a nonedge vessel pixel; therefore, attempts must be made to distinguish the edge from the nonedge vessel pixel. To achieve this, the confidence level of an edge vessel pixel needs to be computed; this was represented by *E* while that of the nonedge vessel pixel was represented by *N*. Subsequently, the edge confidence levels of the LoG filter *m*_1_ were represented by *Em*_1_ while those of canny filter and D-S were represented by *Em*_2_ and *E*_DS_, respectively.

These confidence levels were computed using equations (9), (10), and (11), respectively:

(9) $$\begin{array}{c} Em_{1}=\frac{g\left(x,y\right)}{g_{\max}}, \end{array}$$

(10) $$\begin{array}{c} Em_{2}=\frac{g\left(x,y\right)}{\text{threshold}}, \end{array}$$

(11) $$\begin{array}{c} E_{\text{DS}}=\frac{Em_{1}Em_{2}}{1-Em_{1}Nm_{2}-Nm_{1}Em_{2}}, \end{array}$$

where *Nm*_1_ is the nonedge confidence level for the LoG filter, *Nm*_2_ is the nonedge confidence level for canny edge filter, and the threshold is the max intensity gradient value. The nonedge confidence level for the D-S filter NE_DS_ was calculated using

(12) $$\begin{array}{c} {\text{NE}}_{\text{DS}}=1-E_{\text{DS}}. \end{array}$$

#### 3.3.2. Optic Disk Localization and Exudate Detection and Segmentation

The only bright region in retinal images is the optic disc, and other bright regions are a result of pathological signs which are majorly exudates. Therefore, the first task toward exudate segmentation is to identify and remove the optic disk from the retinal image before classifying other pixels as either exudate or nonexudate pixels. In detecting and removing the optic disk, the morphological component analysis which operates by varying the intensity of image pixels based on an initial threshold was adopted. The algorithm as proposed in [31] is as follows:

Step 1: start
Step 2: input parameters: image *I*_retina_, the set Ф of optic disk pixels (*Ф*_OD_) and exudate pixels (*Ф*_*E*_), the number of expected iterations denoted by *N*_iteration_, and the threshold value *λ*_*v*min_
Step 3: initialization: set number of subpixels *P* = 2, i.e., Ф = [*Ф*_OD_, *Ф*_*E*_], initial optic disk value OD_*v*_=0, and initial exudate value *E*_*v*_=0
Step 4: compute pseudo-subpixel *P*^*∗*^=argmax_*p*_‖*Ф*_*p*_*I*_retina_‖ where *p* = 1, … , *P*
Step 5: set *λ*_*v*_=*λ*_0_=max_*p*≠*P*^*∗*^_‖*Ф*_*p*_*I*_retina_‖
Step 6: iterate *N*_iteration_ times
For the OD components:
  - Update OD_*v*_ when *E*_*v*_ is fixed
  - Calculate the pixel residuals *r* such that *r*=*I*_retina_ − OD_*v*_ − *E*_*v*_
  - Calculate the shift invariant shearlet transform Θ_SIST_ of OD_*v*_+*r* and obtain the coefficient vector *α*_OD_ such that Θ_SIST_ (OD_*v*_+*r*)
  - Hard threshold *α*_*S*_ with *λ*_*v*_ to obtain $\overset{ˇ}{\alpha_{S}}$
  - Reconstruct OD_*v*_ such that OD_*v*_=$\Theta_{\text{SIST}}\overset{ˇ}{\alpha_{\text{OD}}}$
For the exudate components:
  - Update *E*_*v*_ when OD_*v*_ is fixed
  - Calculate the pixel residuals *r* such that *r*=*I*_retina_ − *E*_*v*_ − OD_*v*_
  - Calculate nonsubsampled contourlet transform Θ_NSCT_ of *E*_*v*_+*r* and obtain the coefficient vector *α*_*E*_ such that Θ_NSCT_(*E*_*v*_+*r*)
  - Hard threshold *α*_*E*_ with *λ*_*v*_ to obtain $\overset{ˇ}{\alpha_{E}}$
  - Reconstruct *E*_*v*_ such that *E*_*v*_=$\Theta_{\text{NSCT}}\overset{ˇ}{\alpha_{E}}$
Step 7: update the threshold *λ*_*v*_ =  *λ*_*v*_*x*(*λ*_0_/*pσ*) ^1/(1 − *N*_iteration_)^ where *σ* is the noise standard deviation
Step 8: if *λ*_*v*_>*λ*_*v*min_, go to step 2; else, finish
Step 9: output: morphological components *E*_*v*_and OD_*v*_

The initial threshold *λ*_*v*min_ was computed using equation (13) such that

(13) $$\begin{array}{c} \lambda_{v\min}=\frac{1}{MN}\sum_{x-\left(M/2\right)}^{x+\;\left(M/2\right)}\sum_{y-\left(N/2\right)}^{y+\left(N/2\right)}P\left(x,y\right), \end{array}$$

where *M* and *N* are the row and columns in the bright regions and *P*(*x*, *y*)  is the pixel in the bright region.

Edge distance-seeded region growing method proposed in [32] was adopted to identify the true exudates out of the computed exudate pixels. To achieve this, the initial set of points called seeds were manually determined after which the neighboring pixels were visited in a particular order depending on the chosen similarity criterion. The adopted edge distance-seeded region growing is as follows:

  Step 1: start
  Step 2: input a set of seed pixels *E*_*v*_
  Step 3: choose a window size *M* ×  *N* from the edge image
  Step 4: select a seed pixel *E*_*v*_
  Step 5: select a nonedge and nonseed pixel *P* in the window
  Step 6: compute the distance *PE*_*v*_ between *p*to the nearest seed pixel *E*_*v*_
  Step 7: compute the distance *E*_*v*_*E*_*n*_ between *p*to the nearest seed pixel *E*_*v*_
  Step 8: trace a line *L*that passes between pixels *E*_*v*_and *E*_*n*_
  Step 9: if *PE*_*v*_  <  *E*_*v*_ *E*_*n*_, then the pixel is an edge pixel
  Step 10: include *P* in the segmented output
  Step 11: repeat steps 5 to 10 until there are no more *P*
  Step 13: stop

### 3.4. Feature Extraction

Feature extraction is another important stage in every biometric recognition system as its success will go a long way to determine the output of the next phase (matching/classification phase) and the eventual accuracy of the recognition system [32]. It is the process of generating unique features from the acquired biometric traits. Due to the richness of the retinal blood vessel pattern, it is possible to obtain 400 unique data points from the retina [2]. These features are further used to form a feature vector which makes up a feature template to be stored in a database for recognition purposes. For the training purposes, a total of sixteen features in three categories were extracted from the retinal images. Lesion-based and morphological features were extracted from DR retinal images using connected component analysis and statistical techniques, respectively, while image-based structural features such as luminance, contrast, and structure were computed from healthy retinal images.

#### 3.4.1. Extracting Features from Healthy Retinal Images

Since healthy retinal images are clean and do not have pathological symptoms, structural features that define the relationship between its components were computed. These are luminance, contrast, and structure; they are computed using mathematical formulas highlighted in equations (14), (15), and (16), respectively:

(14) $$\begin{array}{c} \text{luminance feature}\left(f_{1}\right)=\frac{2\mu_{N}+C}{\mu_{N}^{2}}, \end{array}$$

(15) $$\begin{array}{c} \text{contrast feature}\left(f_{2}\right)=\frac{2\sigma_{N}+C}{\sigma_{N}^{2}}, \end{array}$$

(16) $$\begin{array}{c} \text{structure feature}\left(f_{3}\right)=\frac{2\sigma_{N}+C}{\sigma_{N}+C}, \end{array}$$

where *μ*_*N*_ remains the mean intensity of the image, *σ*_*N* _ remains the standard deviation of the intensity, and *C* is a constant used to avoid instability; it was calculated using

(17) $$\begin{array}{c} C={\left(KL\right)}^{2}, \end{array}$$

where *L* is the range of pixel values, i.e., 255 (256-1 for 8-bit grayscale image), and *k* is a value ≪1; 0.01 was preferred after considering different values. Finally, extracted features from the healthy images were saved in a features database for matching purposes.

#### 3.4.2. Extracting Features from Diabetic Retinopathy Retinal Images

Pathological symptoms of diabetic retinopathy are indicated by the presence of exudates and they also cause a great alteration in the vascular patterns of the blood vessels. Therefore, morphological features were extracted from segmented blood vessels while lesion-based features were extracted from segmented exudates.

*(1) Extracting Morphological Features from Segmented Blood Vessels*. Six morphological features were computed and extracted from the segmented blood vessels of diabetic retinopathy images; they are as follows:

(a) Vessel diameters(*f*_4_): this is the mean width of blood vessels in the input retinal image. It is defined as the shortest line segment (Euclidean distance) between two edges *A* and *B* of a blood vessel wall. The Euclidean distance was computed using the following equation:
  - $E_{D}=\sqrt{{\left|X_{A}-\;X_{B}\right|}^{2}+{\left|Y_{A}-Y_{B}\right|}^{2}}$
  - *if* *E*_*D*_ ≥ 11,  it is a main vessel; if 4 ≤ *E*_*D*_ ≤ 7, it is a secondary vessel while if 2 ≤ *E*_*D*_ < 3, it is a vascular vessel
(b) Lines (*f*_5_): these are straight paths between two pixels.
(c) Branch points (*f*_6_): these are points where a pixel originates from.
(d) Ridges end points (*f*_7_): these are where the curved lines of a pixel terminate.
(e) Bifurcation points (*f*_8_): these are points where two ridges come together or where a pixel divides into two branches.
(f) Bifurcations and ridge endings (minutiae) (*f*_9_): these are points where blood vessels divide into two branches and where they terminate.

The connected component analysis proposed in [19] that measures the degree of connectivity of the image pixels called connectivity number *c*_*n*_was used to determine and measure morphological features *f*_5_ − *f*_9_. If *c*_*n*_=1, then, it is a vessel end point; if *c*_*n*_=2, it is a line; if *c*_*n*_=3, it is a bifurcation point; if *c*_*n*_=4, it is a crossover point. If *c*_*n*_=2 & 1, it is a bifurcation end.

*(2) Extracting Lesion-Based Features from the Segmented Exudates*. Finally, seven lesion-based features were extracted from the exudates of the diabetic retinal images using statistical techniques. The features are discussed as follows:

- Extent (*f*_10_): this is the ratio of pixels in the exudate regions to the pixels in the whole image. Extent should have a low value for true exudates detected compared to the false vessel segment.
- Major axis length (*f*_11_): this is the length of the major axis of the exudate regions. The value of the major axis length should be high for exudate regions compared to nonexudate regions.
- Filled area (*f*_12_): this is the number of pixels in the filled image. This should yield a high value for false exudate regions and have more number of pixels in the exudate region than nonexudate regions.
- Mean intensity (*f*_13_): this calculates the mean of all intensity values in the whole image. Nonexudate regions should have a uniform high mean intensity value while exudate regions should have an uneven low mean intensity value.
- Max intensity (*f*_14_): this measures the highest intensities in the whole image. The value should be low for the segmented exudate region while nonexudate regions should have a high value.
- Energy(*f*_15_): this is a measure of the sum of intensity squares of all pixel values within the exudate regions.
- Compactness (*f*_16_): this is a measure of the shape of the exudate regions. It was computed using

(18) $$\begin{array}{c} f_{16}=\;\frac{p^{2}}{4\pi A}, \end{array}$$

  where *A* is the segmented region and *p* is the perimeter of the exudate region.

### 3.5. Matching

This stage helps to determine if an individual's claim to an identity is genuine or false. To achieve this, features extracted from the input retinal images (Query images) were compared with the features stored in the feature template (Stored Templates) using template matching algorithm as proposed in [14]:

  Step1: start
  Step 2: input the query template *Q*_*T*_
  Step 3: fetch the corresponding stored template *S*_*T*_
  Step 4: subdivide each input template into 8 × 8 sized subregions *QT*_*SR*_and *ST*_*SR*_
  Step 5: let the total number of Matched Point (MP) be initialized to 0
  Step 6: compute the intersection point in QT_SR_ and ST_SR_ as IP_QTSR_ and IP_STSR_, respectively
  Step 7: considering QT_SR_, ST_SR_, and IP_QTSR_, compute IP_STSR_ and its subregion that has minimum distance *D*_min_
  Step 8: if *D*_min_  ≤ *D*_threshold_ and IP_QTSR_ have not been matched, then there is an increment in MP. *D*_threshold_ is the maximum offset at which the templates can be displaced
  Step 9: compute Total Matched Points (TMPs)
  Step 10: compute matching percentage *P* of the intersection points such that

(19) $$\begin{array}{c} P=\left(\frac{2*\text{TMP}}{{\text{TIP}}_{\text{QTSR}}+{\text{TIP}}_{\text{STSR}}}\right)*100, \end{array}$$

  where TIP_QTSR_ is the total intersection point in the subregions of the query template and TIP_STSR_ is the total intersection point in the subregions of the stored template
  Step 11: compute matching similarity *S* using

(20) $$\begin{array}{c} S=\max\left\{\text{templatematching}\left(Q_{T},S_{T}\right)\right\},\left.\text{templatematching}\left(Q_{T},S_{T}\right)\right\}. \end{array}$$

  Step 12: stop

**Figure 1:**
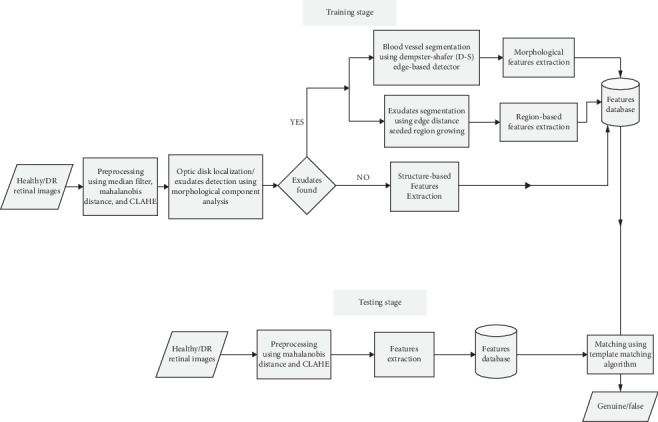
Block diagram of the proposed system.

The maximum value of the matching score was used in determining if *Q*_*T*_ is genuine or from an impostor. If the obtained maximum value is greater than 90%, then the query template is from a genuine individual; else, false. A summary of all these stages is captured in Figure 1.

## 4. Results and Discussion

The results obtained from the evaluation of the proposed recognition system are discussed in this section.

### 4.1. Training the Developed System

After implementing the algorithms proposed at each stage of the research, the developed system was trained with selected healthy as well as DR retinal images. A total of thirty healthy and DR retinal images were randomly selected from DRIVE, HRF, DIARETDB1, and DRiDB databases for the training purpose while sixty images were used for testing. A 30 × 16 feature matrix was produced to connote thirty images used for the training and sixteen features extracted.

### 4.2. Preprocessed Retinal Images

**Figure 2:**
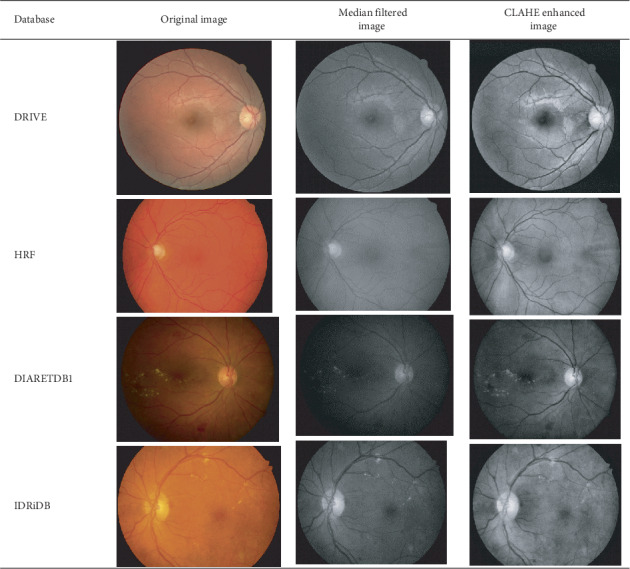
Preprocessed retinal images using the median filter and CLAHE.

All the retinal images used were preprocessed so as to make them suitable for feature extraction. The preprocessing task entails extracting the green component from the input retinal images using Mahalanobis distance, median filter, and Contrast Limited Adaptive Histogram Equalization (CLAHE) techniques. The authors of [28] observed that background images could also introduce some noise into preprocessed images; therefore, they submitted that background images could be extracted for images before preprocessing so that only the foreground image will be extracted. Based on this assertion, Mahalanobis distance was used to separate the background image from the foreground image. The resulting foreground images are then enhanced using median filter and CLAHE as shown in Figure 2. The median filter was used to smoothen and remove noise from the foreground image while CLAHE was used to enhance the contrast of the foreground image. A close physical examination of the preprocessed images shows the differences between the median filter and CLAHE. Retinal blood vessels are not more visible when the median filter was used; however, they became more enhanced when CLAHE was used. Therefore, the preprocessing techniques employed could affect the visibility of the retina components.

### 4.3. Segmented Blood Vessels from the Retinal Images

**Figure 3:**
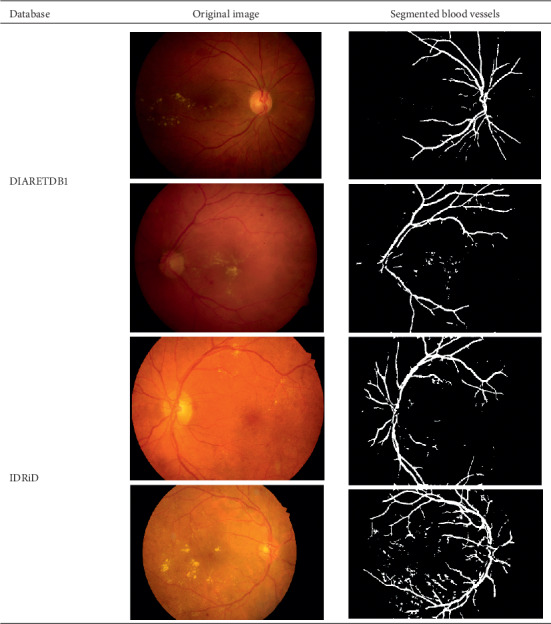
Segmented blood vessels from DR retinal images.

Healthy retinal images have clean components; therefore, their blood vessels were not segmented. However, a major symptom of DR is the alteration of or total damage to the retinal blood vessels' patterns. Therefore, blood vessels were segmented from DR retinal images with a view to extract morphological features from them. A close examination of extracted blood vessels as shown in Figure 3 revealed that most of them are distorted and discontinuous.

### 4.4. Segmented Exudates from the Retinal Images

**Figure 4:**
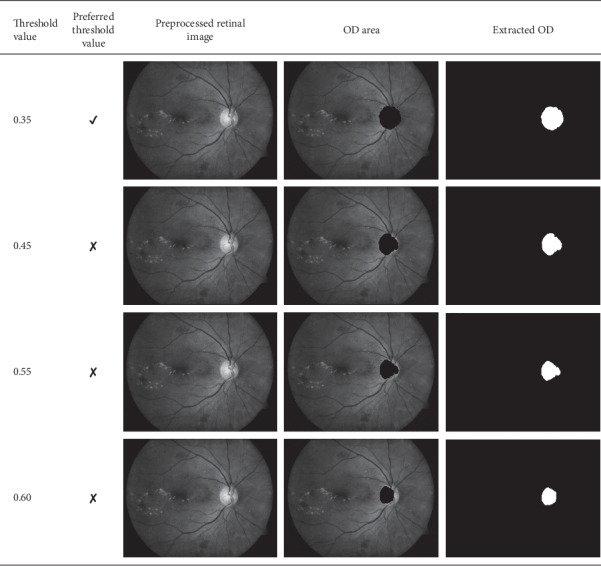
Effects of threshold values on OD localization.

**Figure 5:**
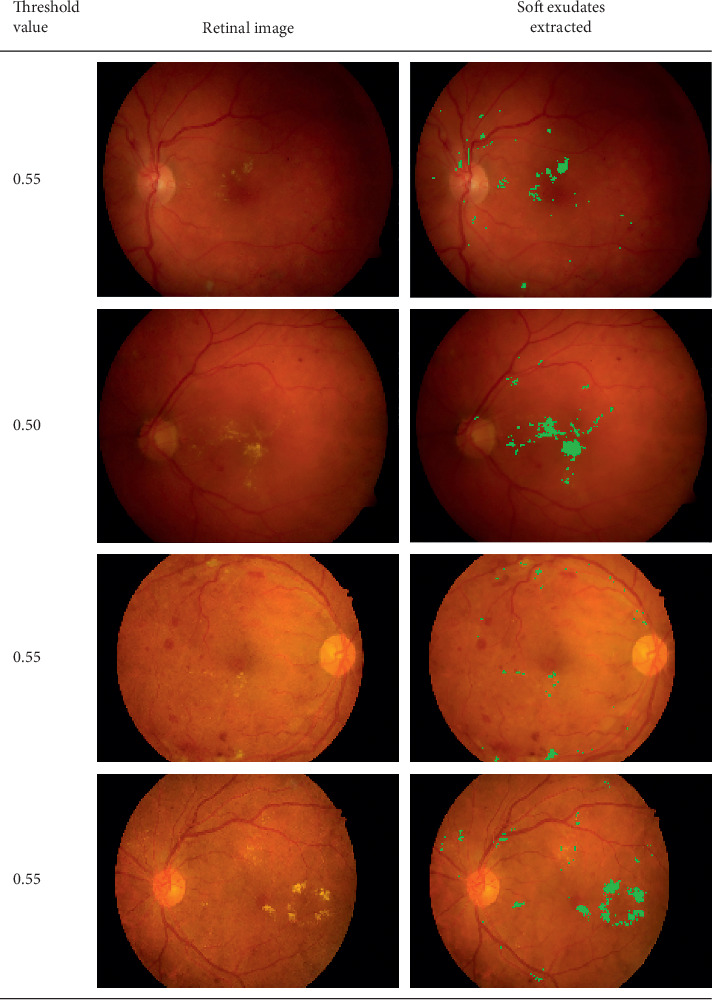
Segmented exudates and their respective threshold values.

The retinal images used for testing have different intensity values; therefore, the threshold value at which the respective OD is detected and extracted differs. However, the threshold values between 0.35 and 0.60 were used. The effect of the various threshold values used and the resulting OD area detected is provided in Figure 4. The soft exudates extracted from the retinal image at different thresholds are provided in Figure 5.

### 4.5. Features Extracted from the Healthy and DR Retinal Images

**Figure 6:**
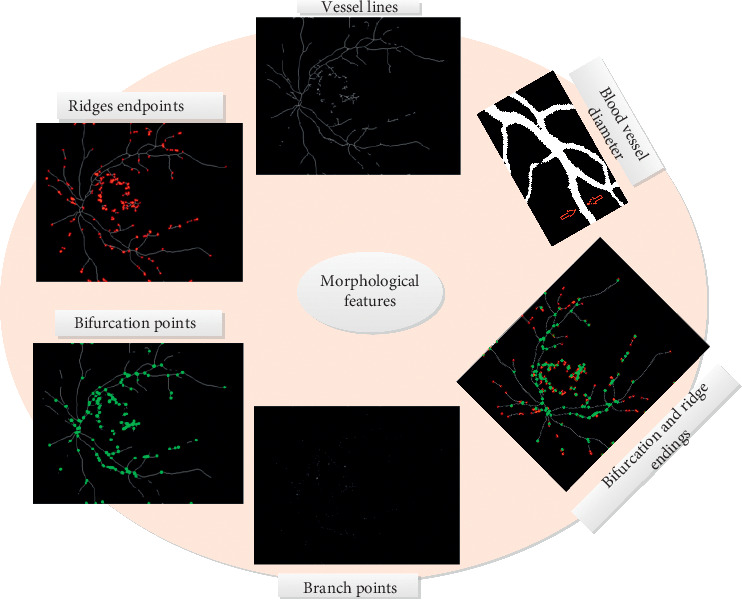
Samples of morphological features extracted from segmented retinal blood vessels.

Three categories of features were extracted from the healthy and DR retinal images. Three structural features, that is, luminance, contrast, and structure, were extracted from the healthy retinal images. These were calculated using statistical formulas.  Table 2 provides some structural features computed from selected healthy retinal images. Moreover, 6 morphological features were extracted from segmented blood vessels. The samples of these are shown in Figure 6.  Table 3 also provides the details of some lesion-based features computed from segmented exudates of DR retinal images. All these features were used to uniquely identify the healthy and DR retinal images.

It was observed that retinal images with more exudates have high values for filled area, extent, and energy. The maximum intensity provides the highest value of intensities computed while the mean intensity provides the mean value of all the intensities computed. How close the exudates are to each other is reflected in the computed value of the compactness. Also, the maximum and mean intensity values of each retinal image were also computed. All these values were used to uniquely identify the DR retinal images.

### 4.6. Performance Evaluation of the Recognition System Using Healthy and DR Retinal Images

The essence of the performance evaluation is to determine if the recognition system will be able to recognize healthy and DR retinal images independently using the proposed framework. The recognition rate (RR), false acceptance rate (FAR), and false rejection rate (FRR) for the two categories of retinal images were, therefore, computed. RR is the percentage of correctly recognized healthy and DR retinal images, FAR is the percentage of wrongly accepted retinal images, and FRR is the percentage of correctly rejected retinal images. These metrics are illustrated with equations (21)–(23). The results obtained are summarized in Table 4:

(21) $$\begin{array}{c} \text{RR}\left(\%\right)=\frac{\text{correctly recognized retinal images}}{\text{total number of retinal images used as a testing set}}=\frac{\text{TP}+\text{TN}}{\text{TP}+\text{TN}+\text{FP}+\text{FN}}, \end{array}$$

(22) $$\begin{array}{c} \text{FAR}\left(\%\right)=\frac{\text{total number of wrongly accepted retinal images}}{\text{total number of retinal images used as a testing set}}=\frac{\text{FP}}{\text{FP}+\text{TN}}, \end{array}$$

(23) $$\begin{array}{c} \text{FRR}\left(\%\right)=\frac{\text{total number of correctly rejected retinal images}}{\text{total number of retinal images used as a testing set}}=\frac{\text{FN}}{\text{FN}+\text{TP}}. \end{array}$$

#### 4.6.1. Recognition Rate of the Developed Recognition System

As presented in Table 4, a 100% recognition rate was achieved with healthy retinal images used to test the developed system. However, a recognition rate of 96.67% and 97.78% was recorded with DIARETDB1 and IDRiDB datasets, respectively.

#### 4.6.2. FAR, FRR, and Equal Error Rate of the Developed Recognition System

**Figure 7:**
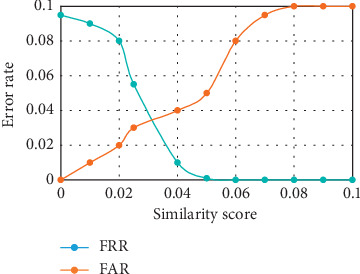
Equal error rate for the DRIVE database.

**Figure 8:**
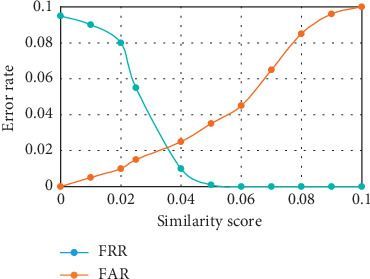
Equal error rate for DIARETDB1.

**Figure 9:**
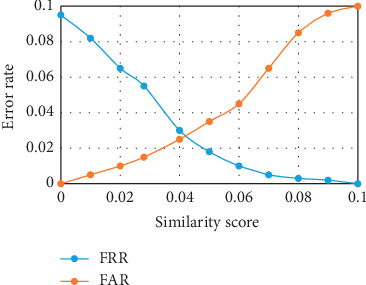
Equal error rate for IDRiD.

The FAR and FRR were computed from the values of the TP, TN, FP, and FN obtained. An overall FAR of 0.0444 and FRR of 0.0133 were achieved. The low value of the FRR is a desirable one. However, a low value for FAR would have been preferred also but certain DR retinal images were not recognized due to the illumination effect on the image which distorted the intensity of the image. To compute the EER value, DRIVE, DIARETDB1, and IDRiD retinal databases were used. For the FAR, the retinal images were divided into groups, and then the ability of the system to accept the impostor retinal image was tested. Likewise, for the FRR, instances where the system rejected retinal images of authorized individuals were measured. As illustrated in Figures 7–9, the EER value of 0.037 at 0.032 similarity score was recorded with the DRIVE database. EER value of 0.021 at 0.038 similarity score was recorded with the DIARETDB1 database while the EER value of 0.025 at 0.042 similarity score was recorded with the IDRiD database. The low values of EER obtained showed that there is a balance between the FAR and the FRR obtained from the system.

## 5. Conclusion

This article has proposed and implemented a framework for the recognition of healthy and DR retinal images. The algorithms employed at each recognition stage were carefully selected to obtain an optimal recognition of healthy as well as DR retinal images. Emphasis was laid on the feature extraction stage since it has a direct effect on the accuracy of biometric recognition systems. With a retina recognition system that can recognize both healthy and DR individuals in view, features extracted from both healthy and DR individuals were used to train the recognition system. As shown in the recognition accuracy obtained, the ability of the developed system to identify healthy and DR retinal images affirmed the proposition that if features extracted from healthy and DR retinal images were used to train a retinal recognition system, then such biometric system should be able to accommodate any possible future changes in the features of healthy retinal images used to train a retinal recognition system.

## Figures and Tables

**Table 1:** Summary of retinal images used.

S/N	Retinal database	Healthy retinal images	DR retinal images	Total	Dimension	Format
1	DRIVE	30		30	768 × 584	JPEG
2	HRF	15	15	30	3504 × 2336	JPEG
3	iDRiD	—	45	45	4288 × 2848	BMP
4	DiaretDB1	—	30	30	1500 × 1152	PNG
	Total	45	90	135		

**Table 2:** Structural features computed from selected healthy retinal images.

S/N	Retinal image	Luminance	Contrast	Structure
1.	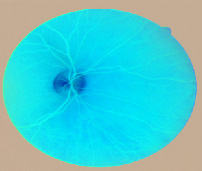	0.032173	0.030270	1.91408
2.	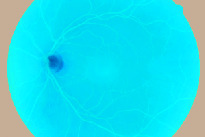	0.024717	0.024565	1.92858
3.	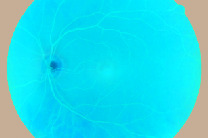	0.025434	0.025819	1.92533
4.	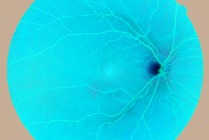	0.024093	0.027797	1.92028

**Table 3:** Lesion-based features extracted from segmented exudates.

S/N	Lesion-based features/retinal image	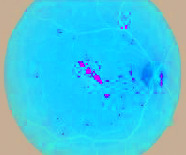	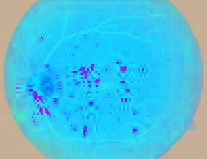	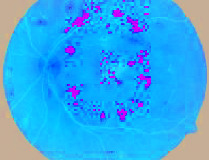
1	Extent	0.4944	0.5802	0.7500
2	Major axis length	6.2416	5.27689	0.9765
3	Filled area	15	25	47
4	Energy	1103	2164	2828
5	Compactness	0.7156	1.0119	0.6477
6	Max intensity	0.6328	0.6356	0.7657
7	Mean intensity	0.2495	0.2296	0.3062

**Table 4:** Recognition rate of the developed recognition system.

S/N	Retinal images (total)	Database (total)	RR (%)
1	Healthy (45)	DRIVE (30)	100
		HRF (15)	100
2	Diabetic retinopathy (75)	DIARETDB1(30)	96.67
		IDRiDB (45)	97.78

## Data Availability

The retinal blood vessel data used to support the findings of this study have been deposited in http://doi.org/10.5281/zenodo.1410542 and http://doi.org/10.5281/zenodo.1409114 for academic and research purposes.
